# AI-powered generative mRNA design: GEMORNA enables efficient expression and delivery for next-generation RNA therapeutics

**DOI:** 10.1016/j.jpha.2026.101671

**Published:** 2026-05-26

**Authors:** Yan Chen, Quan Dai, Li Ye

**Affiliations:** a State Key Laboratory of Quality Research in Chinese Medicines and Faculty of Chinese Medicine, Macau University of Science and Technology, Macao SAR, 999078, China; b Department of Pharmacy, Center for Translational Research in Cancer, Sichuan Clinical Research Center for Cancer, Sichuan Cancer Hospital & Institute, Affiliated Cancer Hospital of University of Electronic Science and Technology of China, Chengdu, 610000, China

**Keywords:** mRNA design, Artificial intelligence, Cell therapy

## Abstract

• This manuscript introduced GEMORNA, a comprehensive platform leveraging deep generative AI to autonomously design highly optimized, full-length mRNA and circular RNA sequences.
• This represents a significant paradigm shift from traditional, often manual and piecemeal, RNA optimization strategies.
• GEMORNA-generated sequences achieve remarkable enhancements in protein expression, stability, and delivery efficiency, while simultaneously mitigating immunogenicity—a major hurdle in RNA therapeutics.

## Graphical abstract

**Figure:**
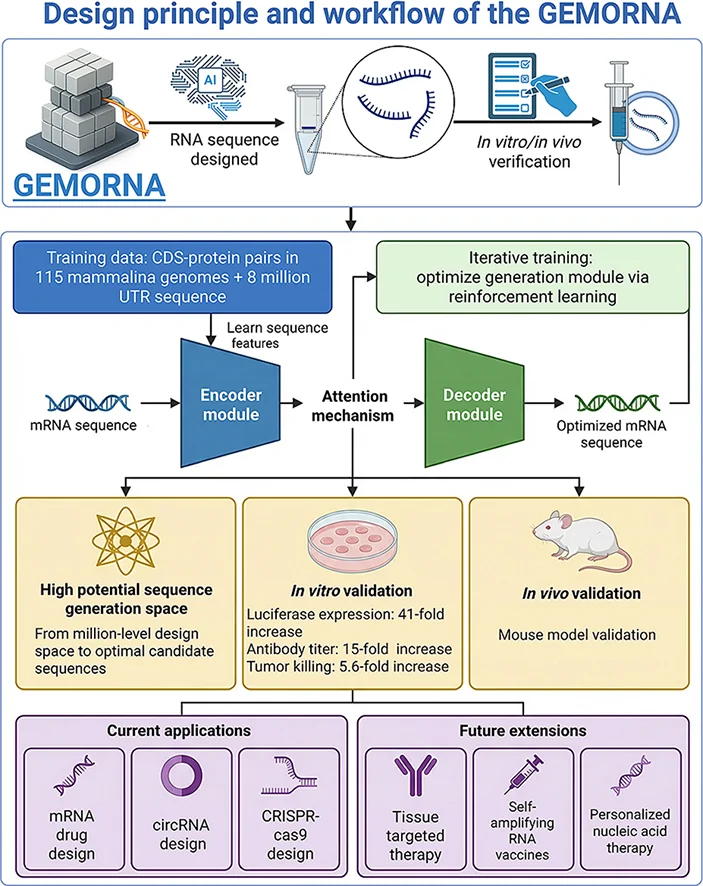

In recent years, nucleic acid-based therapeutics such as messenger RNA (mRNA) have emerged as the forefront of research in the fields of vaccines and protein replacement therapies, owing to their straightforward sequence design and highly programmable biological functions. However, traditional mRNA design approaches have often been constrained by bottlenecks such as low expression efficiency, insufficient stability, and high immunogenicity [1]. To address these challenges, the Generative Models for RNA (GEMORNA) platform leverages generative artificial intelligence models driven by deep learning to automatically generate high-activity, high-stability full-length mRNA and circular RNA (circRNA) sequences (Fig. 1). The GEMORNA platform consists of a generative module based on a Transformer encoder-decoder architecture and an evaluation module that predicts expression levels and immunogenicity scores. The generative module learns the distribution of high-performance RNA sequences from training data, while the evaluation module provides real-time feedback on the quality of generated sequences. Currently, the platform has been validated for conventional mRNA and circRNA. While the generative framework holds potential for other modalities—such as self-amplifying RNA (saRNA) or clustered regularly interspaced short palindromic repeats (CRISPR) guide RNAs (gRNAs)—these applications would require specific model retraining and are subjects for future exploration (Fig. 1).

**Fig. 1:**
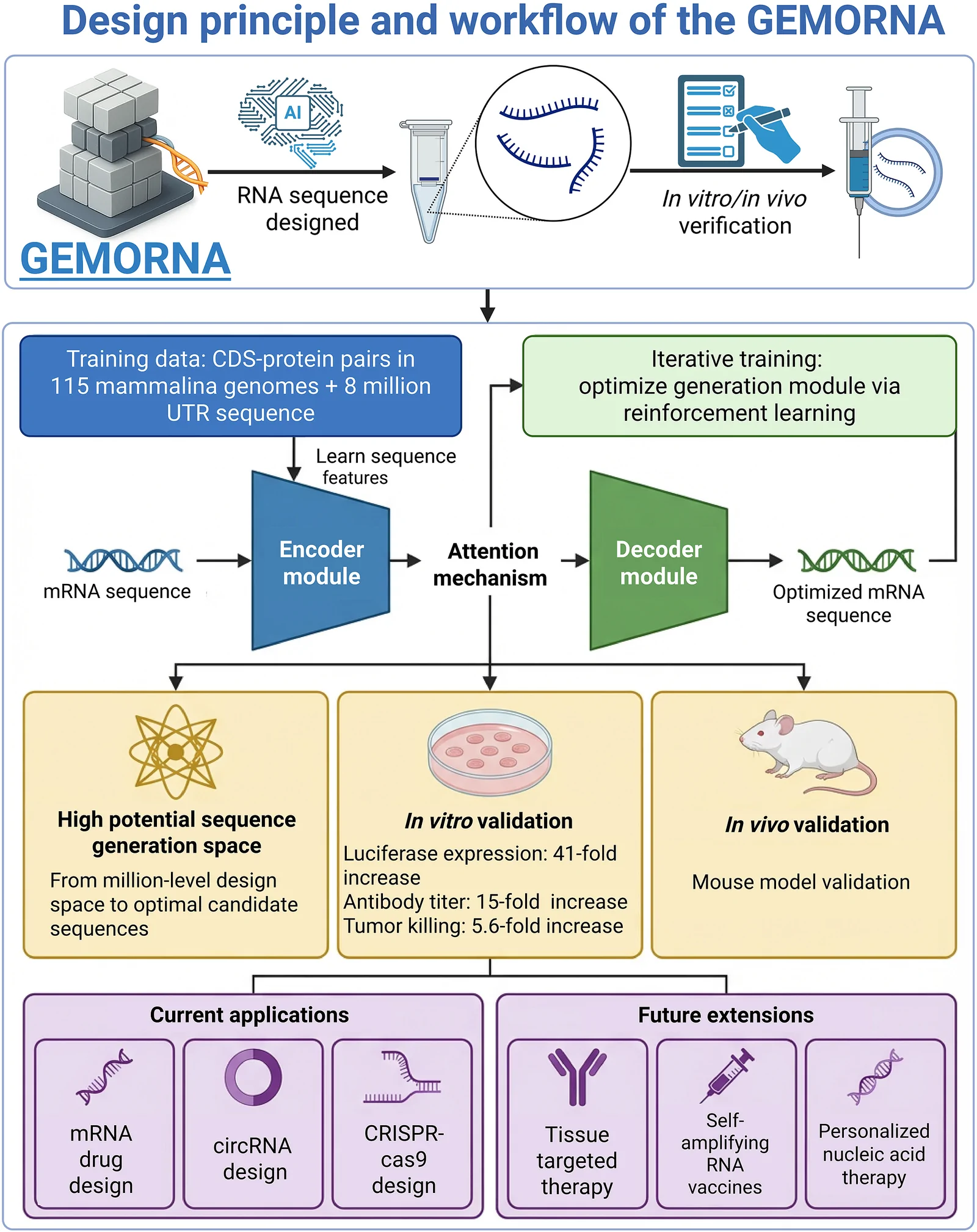
Design principles, workflow, and applications of the Generative Models for RNA (GEMORNA) platform. The schematic illustrates the deep learning-driven framework of GEMORNA for optimizing RNA therapeutics. The overall workflow integrates artificial intelligence (AI)-based sequence design with wet-lab validation (*in vitro* and *in vivo*). Then, the core architecture utilizes a transformer-based dual-module system. The model is trained on a massive dataset comprising over 1 million mammalian coding sequence (CDS)-protein pairs and 8 million untranslated region (UTR) sequences. The encoder module extracts sequence features, while the decoder module works with an attention mechanism to generate optimized messenger RNA (mRNA) sequences. An iterative training loop employing reinforcement learning further refines the generation module based on evaluation scores. The platform's efficacy is validated through high-potential sequence generation and rigorous testing. *In vitro* validation shows a 41-fold increase in luciferase expression, and *in vivo* validation demonstrates significant therapeutic efficacy. Current applications cover mRNA drugs and circular RNA (circRNA) design, with future extensions planned for tissue-targeted therapies, self-amplifying RNAs (saRNAs), and personalized nucleic acid therapies. CRISPR: clustered regularly interspaced short palindromic repeats; Cas9: CRISPR-associated protein 9.

The artificial intelligence (AI)-designed sequences were subsequently synthesized via *in vitro* transcription (IVT), encapsulated into lipid nanoparticles (LNPs), and rigorously validated in both cellular and animal models. By integrating these outputs with optimized delivery systems, the platform significantly enhances protein expression both *in vitro* and *in vivo*, paving the way for transformative applications in nucleic acid therapeutics and cell-based therapies. The most notable innovation of GEMORNA lies in its adoption of deep generative architectures, exemplified by the transformer model, to optimize RNA sequences. The present study validates GEMORNA's efficacy for designing conventional mRNA and circRNA. The platform not only automates the design of coding sequence (CDS) but also excels in functional prediction and optimization of regulatory regions, such as 5′ and 3′ untranslated regions (UTRs), thereby achieving efficient global expression at the molecular level. Powered by a data-driven system, GEMORNA learns hidden encoding principles that govern the correlation between sequence attributes and expression levels by analyzing large-scale biological information databases, expression profiles, and structural data. Unlike traditional approaches that rely on manual adjustments or single algorithms, GEMORNA can rapidly produce RNA molecules tailored to diverse targets and scenarios, offering a robust technological foundation for personalized medicine and the treatment of complex diseases.

In experimental validations, the mRNA sequences generated by GEMORNA demonstrated exceptional expression capabilities in both *in vitro* and *in vivo* settings. To minimize experimental variability and ensure data simultaneity, all high-throughput screenings were conducted under standardized conditions with synchronized time points and consistent culture volumes. For example, when tested using luciferase reporter genes, the mRNA sequences designed by GEMORNA displayed expression levels in HEK293T cells that were up to 41-fold higher than those achieved using widely used benchmarks, including commercial codon optimization tools (e.g., GenScript, integrated DNA technologies (IDT)), state-of-the-art algorithms like LinearDesign and biLSTM-CRF, and standard sequences from commercial vectors (pGL4.11) [2]. The iterative feedback loop involved multiple cycles of AI design, experimental validation, and model refinement. Experimental validation was performed under standardized conditions: HEK293T and HeLa cells were cultured in dulbecco's modified eagle medium (DMEM) supplemented with 10% fetal bovine serum (FBS) at 37 °C with 5% CO_2_. Cells were transfected with LNP-encapsulated mRNA at a concentration of 50 ng/well in 96-well plates. Protein expression was measured 24–48 h post-transfection using luciferase assays or fluorescence-based methods. The resulting data were used to retrain the AI model, improving its predictive accuracy with each iteration. For therapeutic proteins such as erythropoietin (EPO), mRNA sequences delivered via LNPs showed expression levels over 15 times higher than the control group. Similarly, animal model experiments demonstrated a significant improvement in protein expression and prolonged expression duration for GEMORNA-generated mRNA, underscoring the platform's utility in preclinical research. Data robustness was ensured through multiple biological replicates (*n* = 3 or *n* = 4) and rigorous statistical analyses, such as one-way analysis of variance (ANOVA), providing strong evidence for the reliability of the conclusions. The delivery system of the GEMORNA platform also achieved innovative breakthroughs. By deeply integrating with advanced LNP technologies, the platform achieved high RNA encapsulation efficiency, improved molecular stability, and excellent biocompatibility. RNA synthesis followed established IVT protocols, including the incorporation of a standard 5′ cap structure (e.g., CleanCap) and a poly(A) tail of defined length, to ensure proper maturation and stability.

The resulting LNP formulations were characterized for particle size and distribution using dynamic light scattering (DLS) and for RNA encapsulation efficiency using high-throughput fluorescence quantification (e.g., RiboGreen assay). Only batches meeting stringent quality criteria proceeded to *in vitro* and *in vivo* testing for quality control. Quality control was strictly enforced, only batches meeting stringent criteria (e.g., encapsulation efficiency > 90%, PDI < 0.2) proceeded to biological testing. Particle characterization utilized DLS with a 633 nm laser source to analyze scattering patterns, ensuring the LNPs maintained a uniform size distribution (80–120 nm), consistent with established principles of nanoparticle optics [[3], [4], [5]].

For multi-gene co-expression studies, GEMORNA-designed mRNAs for different targets were produced individually, mixed at predetermined ratios, and then co-encapsulated into LNPs for delivery. This ensured stable RNA concentration and controllable expression post-delivery, addressing the longstanding challenge of balancing efficiency and safety in conventional delivery systems. Systematic analyses of "protein expression peak distribution" revealed that GEMORNA consistently outperformed mainstream designs across various cell lines and multiple organ distributions *in vivo*. Notably, the platform supports the co-expression of multiple genes and the design of circRNAs. Functional screening studies confirmed that mRNA generated by GEMORNA could simultaneously express multiple target proteins in single-cell or animal models while maintaining expression uniformity and stability. This feature greatly expands the potential applications of nucleic acid therapeutics in combination therapies, complex trait screening, and functional genomics. For instance, in chimeric antigen receptor T-cell (CAR-T cell) therapy experiments, circRNA designed by GEMORNA delivered to T cells significantly enhanced their cytotoxic activity and anti-tumor efficacy compared to conventional circRNA designs, providing a new avenue for cell-based therapy and personalized cancer immunotherapy.

Regarding immunogenicity and safety, the GEMORNA platform supports nucleotide modifications, such as m1Ψ, to reduce innate immune activation and inflammation risk at the sequence level. Both *in vitro* and *in vivo* results demonstrated that optimized mRNA sequences induced minimal abnormal immune responses in animal models, displayed sustained protein expression, and significantly reduced off-target effects, laying a solid foundation for large-scale clinical applications of nucleic acid-based therapeutics in the future. High-throughput molecular detection and stringent experimental design underpin the scientific rigor of the GEMORNA platform. Experimental techniques such as nanoscale fluorescence detection, enzyme-linked immunosorbent assay (ELISA)-based protein quantification, and live-animal imaging, combined with multiple controls and biological replicates, have ensured the reliability and reproducibility of the findings. For example, in an EPO time-course expression study in mice, GEMORNA mRNA yielded not only higher peak expression levels but also an extended duration of expression—nearly double that of the control—demonstrating its consistent therapeutic potential *in vivo*. Moreover, the platform facilitates large-scale, automated functional gene screening, enabling research teams to quickly design and validate hundreds to thousands of target sequences, thereby accelerating the discovery of new therapeutic targets, drug candidates, and disease mechanisms. By combining high-throughput delivery with molecular detection, GEMORNA significantly reduces the time required to transition from basic research to application development for personalized mRNA therapeutics. It is worth noting that while physical factors in the experimental setup—such as vessel curvature or external fields—could theoretically influence signal acquisition, the use of strictly standardized protocols and appropriate biological controls in this study minimized such interference, ensuring that the observed performance gains were attributable to the AI-optimized sequences.

Looking ahead, the GEMORNA platform holds promise for empowering mRNA and circRNA therapeutics while offering robust tools for vaccine development, protein replacement therapies, and the treatment of genetic disorders. Currently, the platform has been successfully adapted to various applications, including the design of antigens for COVID-19 vaccines, expression of EPO, tumor immunotherapy, and tri-gene co-activation. For instance, in a COVID-19 vaccine project, the neutralizing antibody titers generated by mRNA sequences produced by GEMORNA surpassed those of mainstream mRNA vaccines by 15%–30%, demonstrating its breakthrough potential for immunotherapeutic nucleic acid-based drugs. By integrating generative AI models with iterative feedback from experimental validation, GEMORNA enables automated, high-throughput, and intelligent optimization, driving advancements in personalized nucleic acid therapies and precision medicine. Its low immunogenicity, high expression efficiency, and broad adaptability address key technical bottlenecks of traditional mRNA drugs while unlocking new possibilities for clinical trials and large-scale applications. According to published data, GEMORNA consistently outperforms mainstream algorithm-based sequences in both peak protein expression and sustained expression duration, firmly establishing itself as a core innovation engine for next-generation RNA drug development. The generative AI architecture of GEMORNA is inherently adaptable and holds promise for designing other RNA modalities, such as saRNA or gRNAs for gene editing systems like clustered regularly interspaced short palindromic repeats-CRISPR-associated protein 9 (CRISPR-Cas9). However, this would require targeted model training and experimental validation, representing a promising direction for future work.

In summary, the GEMORNA platform achieves unprecedented improvements in RNA expression efficiency and safety through AI-driven generative sequence design. Its advancements in delivery systems, functional screening, and immunomodulation make it a transformative tool for basic life sciences research and the industrialization of nucleic acid therapeutics. As the platform continues to evolve, its applications in clinical precision therapies, vaccine development, and cell and gene therapy are expected to grow, paving the way for nucleic acid drugs to play an increasingly pivotal role in the future of medicine.

## CRediT authorship contribution statement

**Yan Chen:** Methodology, Writing - original draft, Visualization. **Quan Dai:** Formal analysis. **Li Ye:** Writing - review & editing.

## Declaration of Generative AI and AI-assisted technologies

In the preparation of this manuscript, the authors utilized a large language model, Google AI Studio—Gemini 2.5. This AI-assisted technology was primarily employed to refine language, enhance readability, ensure an appropriate academic tone, and improve the scientific precision of the text. All content generated or assisted by AI was subjected to rigorous oversight, thorough review, and critical editing by the authors. The authors take full responsibility for the accuracy, originality, and integrity of the content presented in this publication.

## Declaration of competing interest

The authors declare that they have no known competing financial interests or personal relationships that could have appeared to influence the work reported in this paper.
